# Advancing Crystallography Research: The Latest Progress and Future Directions of NE-CAT Beamlines at the Advanced Photon Source

**DOI:** 10.1063/4.0001123

**Published:** 2025-10-27

**Authors:** Ali Kaya, Malcolm Capel, Igor Kourinov, Anthony Lynch, Anne Mulichak, David Neau, Kay Perry, Cyndi Salbego, Jonathan Schuermann, Narayanasami Sukumar, Graeme Winter, James Withrow, Frank Murphy

**Affiliations:** Cornell University, Lemont, IL, USA

## Abstract

The Northeastern Collaborative Access Team (NE-CAT) develops and operates advanced synchrotron X-ray beamlines to support high-impact structural biology research. With specialized instrumentation and deep expertise, NE-CAT serves a global user base, facilitating structural determination of challenging macromolecular systems.

Following the completion of the Advanced Photon Source (APS) upgrade, NE-CAT has implemented key improvements to ensure optimal performance, flexibility, and reliability in data collection. These include a **high- speed storage infrastructure** and the deployment of **RAPDv2**—a next-generation data processing suite that offers seamless integration, scalable performance, and automated workflows for molecular replacement (MR) and single- wavelength anomalous dispersion (SAD) experiments.

Ongoing developments include the installation of a **new monochromator** for beamline 24-ID-C, the integration of **MD3 micro-diffractometers** for enhanced precision, and the deployment of a **custom-built automounter** capable of holding 30 sample pucks. Additionally, we are introducing an **upgraded remote user interface** to enable fully automated data collection and expanding support for room-temperature data acquisition experiments.

Currently, NE-CAT operates **beamline 24-ID-E**, a fixed-energy (12.662 keV) undulator beamline optimized for remote use. It’s equipped with an MD2 micro-diffractometer, ALS-style 14-puck automounters, and the RAPDv2 platform. NE-CAT offers **24/7 user support** and secure, flexible data access through **Globus** or **a custom Python sync** script. Beamline 24-ID-C is under development and will soon launch with fully redesigned systems and enhanced capabilities.

NE-CAT is funded by a P30 grant from the National Institute of General Medical Sciences (NIGMS), with additional support from member institutions. We welcome collaboration and community input as we continue advancing resources to meet the evolving needs of structural biology.

